# Molecular carcinogenesis of hepatocellular carcinoma and intrahepatic cholangiocarcinoma: one step closer to personalized medicine?

**DOI:** 10.1186/2045-3701-1-5

**Published:** 2011-01-24

**Authors:** Mia Kumar, Xuelian Zhao, Xin Wei Wang

**Affiliations:** 1 Liver Carcinogenesis Section, Laboratory of Human Carcinogenesis, Center for Cancer Research, National Cancer Institute, Bethesda, Maryland 20892, USA

## Abstract

Hepatocellular carcinoma (HCC) and intrahepatic cholangiocarcinoma (ICC) are the two major forms of primary liver cancers (PLC), accounting for approximately 90% and 5% respectively. The incidence of each is increasing rapidly in the western world, however our knowledge of the underlying mechanisms remains limited and the outcome, dismal. The etiologies of each vary geographically; nevertheless, chronic inflammation has been identified in more than 80% of the cases and appears to be a key mediator in altering the liver microenvironment, increasing the risk of carcinogenesis. However, since not all HCC and especially ICC cases have a recognized risk factor, there are currently two proposed models for liver carcinogenesis. The clonal evolution model demonstrates a multi-step process of tumor development from precancerous lesions to metastatic carcinoma, arising from the accumulation of genetic and epigenetic changes in a cell in the setting of chronic inflammation. While the majority of cases do occur as a consequence of chronic inflammation, most individuals with chronic infection do not develop PLC, suggesting the involvement of individual genetic and environmental factors. Further, since hepatocytes and cholangiocytes both have regenerative potential and arise from the same bi-potential progenitor cell, the more recently proposed cancer stem cell model is gaining its due attention. The integration of these models and the constant improvement in molecular profiling platforms is enabling a broader understanding of the mechanisms underlying these two devastating malignancies, perhaps moving us closer to a new world of molecularly-informed personalized medicine.

## Introduction

Primary liver cancer (PLC) is the fifth most common cancer worldwide and the third most deadly, with approximately 600,000 deaths annually. Hepatocellular carcinoma (HCC), a primary malignancy of the hepatocyte, accounts for approximately 85% to 90% of all PLC, out of which 80% of HCC cases occur in either sub-Saharan Africa or in eastern Asia [1,2]. Cholangiocarcinoma (CCA), a malignancy of cholangiocytes in the biliary epithelium, is the second most common form and accounts for about 5% to 10% of PLC. CCA is categorized as intrahepatic or extrahepatic according to the anatomic location of the tumor. Since intrahepatic cholangiocarcinomas (ICC) usually present in small biliary ducts or ductules they are considered a PLC, compared to extrahepatic cholangiocarcinomas which are a form of biliary tract cancer. The incidence of CCA varies with regards to the two forms; however, for purposes of this review we will address ICC, which has the highest incidence in eastern Asia, particularly Thailand, with an increasing risk ratio in the western world [3-5]. The remaining PLC subtypes, which account for less than 5% of cases, are fibrolamellar HCC, hepatoblastoma, angiosarcoma, epithelioid hemangioendothelioma and hepatocellular adenoma.

Risk factors that lead to the multistep development of HCC are well known and it is established that approximately 80% of HCC cases develop in individuals suffering from chronic hepatitis B or C viral infection (HBV or HCV), cirrhosis, and also those with a high exposure to aflatoxin-b1 (AFB). HCC is particularly attributed to these exposures due to the extensive oxidative stress and release of inflammatory cytokines induced by viral infection in the setting of liver inflammation. Diabetes, obesity, smoking and alcohol abuse have also been associated with the development of HCC, but with reduced frequency [6-8]. Currently, individuals at risk for HCC are routinely screened by ultrasonography and alpha-fetoprotein (AFP) levels but most patients are still diagnosed with an advanced disease stage and therefore a 5-year survival for the majority of HCC patients' remains dismal [9,10]. Due to the high variability in AFP evaluation, affected by the specificity of the test, ethnic backgrounds and tumor size, an improvement in screening procedures is highly awaited. Furthermore, the impairment of liver function and the expression of multi-drug resistance genes render HCC treatment difficult [11]. This review discusses the mechanistic changes that occur during the development of HCC and the potential targets that are being investigated for new screening techniques, with the hope that this will lead to a more personalized treatment regimen to improve patient outcome.

Risk factors for ICC, on the other hand, are not so well established given that approximately 90% of ICC patients lack a recognized risk factor for the disease [12]. Furthermore, ICC cases appear to develop in otherwise healthy livers, with only 10% resulting from chronic inflammation [3]. Nevertheless, relatively strong CCA associations have been established with primary sclerosing cholangitis (PSC) [13-15], liver fluke infestations [14,16] and hepatolithiasis [3]. Other possible, but not well characterized associations may also exist with HBV or HCV infection [17-19] and alcohol consumption [20,21]. The molecular interactions and genomic alterations in cholangiocytes that drive the development of CCA are not clear and the absence of specific symptoms and diagnostic tests make the disease difficult to identify in premalignant stages. Currently, 5-year survival rate for ICC cases remains below 5% [3], with the only hope for improved survival being complete resection of the tumor. This underscores the need for increased research in this field and for the development of improved diagnostic criteria and treatment options. The increased utilization of genome-wide association studies (GWAS) will be particularly useful to identify large-scale genetic variants in PLCs, particularly cholangiocarcinoma, since so little is currently understood.

As mentioned, in this review we will describe the current understanding in the molecular mechanisms, specifically the genetic, epigenetic and signaling alterations that take place, which give rise to the majority of PLC cases observed, separated by HCC and ICC. Furthermore, it is interesting to note the two models for tumorigenesis, a step-wise clonal evolution model and a cancer stem cell (CSC) model, that have arisen as a consequence of our improved understanding in this field. How these models will impact PLC cases in the clinical setting will provide for a stimulating discussion.

## Hepatocellular Carcinoma

### Altered signaling pathways in HCC at the genomic and transcriptomic levels

In a setting of chronic inflammation, the organ microenvironment experiences a variety of molecular changes that, in fact, often stem from the process and consequence of inflammation. In liver, cytokines and reactive oxygen and nitrogen species produced by inflammatory cells have been shown to mediate liver damage and induce the liver's regenerative response [22-26]. This predisposes the proliferating cell to a variety of genetic changes at the genomic and transcriptional levels.

#### Genomic alterations

Large-scale quantitative comparisons of HCC tumors to non-tumors by the use of comparative genomic hybridization (CGH) arrays and loss of heterozygosity (LOH) has revealed the occurrence of chromosomal and microsatellite instability in HCC. The most frequently deleted chromosomes arms are 1p, 4q, 6q, 8p, 9p, 13q, 16p, 16q and 17p and regional gains are most often observed in 1q, 6p, 8q and 17q [27,28], which, in general corresponds to autosome arms that contain allelic deletions identified by LOH: 1p, 1q, 4q, 5q, 6q, 8p, 9p, 13q, 16p, 16q and 17p [27,29,30]. Unrelated to tumor size, individual HCCs can represent multiple allelic deletions and chromosomal gains and losses, which can accumulate during successive cell proliferation events and results in a heterogeneous mixture of genomic aberrations [31]. The heterogeneity of tumors can help to identify tumor origin and due to the sensitivity of CGH and SNP arrays, genomic alterations can be used as fingerprints to identify whether a tumor is a recurrent event or a second primary tumor [32,33]. The frequent loss of chromosome regions observed by LOH and SNP arrays has revealed the concomitant loss or mutation of tumor suppressor genes such as *TP53 *(p53), retinoblastoma *RB1 *(Rb) [34,35], *CDKN2A *(p16^INK4A^) [29,36] and insulin-like growth factor-2 receptor *IGF-2R *[37,38], which are strongly associated with carcinogenetic signaling pathways [29,34,39,40]. Gain of function mutations have also been observed in HCC, for example mutations in CTNNBI (β-catenin), which results in the deregulation of similar signaling pathways in HCC [41,42].

***TP53 ***gene encodes the p53 protein which plays a pivotal role in the DNA-damage response network, including cell cycle arrest, apoptosis, DNA repair and cellular senescence. Therefore, it is not surprising that *TP53 *loss of function mutations or allelic deletions in chromosome 17p are commonly associated with human carcinogenesis [43], and depending on the extent of damage, p53 can either regulate the production of anti-oxidant genes to initiate DNA repair, or induce apoptosis through the activation of pro-oxidant genes[29]. AFB1 is a particular mutagen of *TP53*, causing G:C to T:A transversions at the third base in codon 249 (converting arginine to serine), and the rate of *TP53 *R249S mutation may be accelerated in the presence of viral infection[44,45]. HBV encodes a viral protein, HBx, which can specifically bind to p53 and suppress p53-induced apoptosis [46]. Strong associations have been observed between *TP53 *R249S mutation levels and HCC risk, especially with respect to primary tumor development and also the interval between surgical resection and recurrence [47,48]. A recent study has linked this p53 hotspot mutation to HCC with aggressive tumors, poor prognosis and an acquisition of stem cell-like traits[49], which is not unexpected since a separate study has shown that *TP53 *mutations have the ability to reprogram terminally differentiated cells into pluripotent stem cells[50].

#### Transcriptomic alterations: the deregulation of signaling pathways in HCC

**Figure 1:**
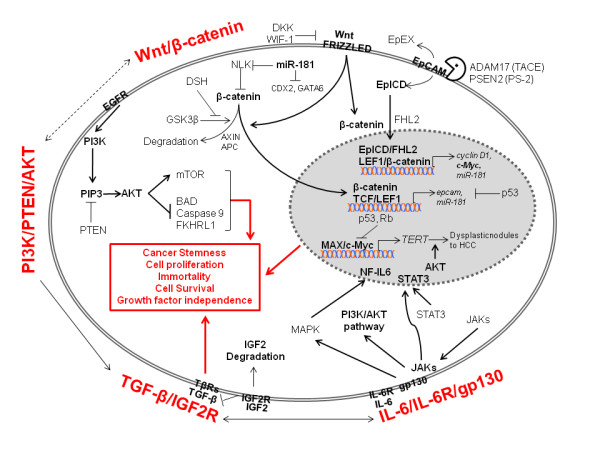
**Signaling Pathways Altered in Hepatic Cancer Stem Cells**. Wnt/β-catenin, PI3K/PTEN/AKT, TGF-β/IGF-2R and IL-6/IL-6R/gp130 signaling pathways have been shown to be activated in HCC. Activation of the Wnt pathway results in β-catenin accumulation in the cytosol and translocation into the nucleus, where β-catenin forms two major protein-DNA complexes. 1) β-catenin/TCF/LEF is a classic complex of Wnt/β-catenin pathway that mediates Wnt target genes expression, e.g. EpCAM and miR-181; 2) EpICD/FHL2/β-catenin/LEF1-DNA complex represents a cross-talk of Wnt/β-catenin with EpCAM signaling pathway [133]. Cleavage of EpCAM by TACE and PS-2 releases EpICD into cytosol which in turn translocates into the nuclues with β-catenin and FHL2, where EpICD/FHL2/β-catenin forms protein-DNA complex with LEF1and regulates EpCAM target genes expression, e.g. cyclin D1, c-Myc andd miR-181. AKT is activated by two phosphorylation sitess Thr308 and Ser473. Phosphorylation of Thr308 is promoted by PI3K and suppressed by PTEN. Activated AKT induces cell survival through the suppressive phosphorylation of BAD and Caspase 9, two apoptosis mediators in unphophorylated status. AKT also acts as a cell cycle progression regulator through activating the mTOR pathway [134]. Two oncogenic pathways PI3K/PTEN/AKT and Wnt/β-catenin may be interconnected to promote stemness and carcinogenesis. Loss of IGF-2R impacts cell proliferation by accumulating IGF-2 mitogen and activation of TGF-β signaling.

Structural genomic mutations and epigenetic changes may lead to altered gene expression patterns that significantly affect the signal transduction pathways in HCC and the variability in pathway expressed may allude to the cellular origin of HCC. A selection of the relevant signaling pathways altered in HCC is discussed here (Figure 1).

**TGF-β **is an inflammatory cytokine implicated in an array of functions such as cell growth, differentiation, migration, apoptosis, adhesion, survival and immunity [51]. IGF-2R, a tumor suppressor gene, promotes the degradation of mitogen IGF-2 and also the simultaneous activation of transforming growth factor-β (TGF-β) signaling, thereby halting cell proliferation and carcinogenesis [52]. Inflammation and subsequent genomic mutations in IGF-2R result in IGF-2 over-expression and a reduction in the inhibitory effects of TGF-β signaling, a feature commonly observed early in the development of HCC [53,54]. Immunohistochemical analysis of HCC has also revealed a disruption of TGF-β signaling coinciding with an increase in the expression of stem cell markers and the activation of interleukin-6 (IL-6). This indicates a link between IL-6, a major stem cell signaling pathway and the disruption of TGF-β signaling, resulting in CSC driven HCC[55].

Interestingly, IL-6 activation is a frequent event in HCC. Recent studies indicate that gain of function mutations of glycoprotein-130 (gp130), a co-receptor of IL-6, is associated with a marked activation of IL-6 in inflammatory hepatocellular adenomas[56]. Noticeably, rare gp130 alterations are always accompanied by β-catenin activating mutations in HCC, suggesting that these two signaling pathways are converged to contribute to hepatocarcinogenesis. Additional details about β-catenin involvement in HCC are described below.

**Wnt/β-catenin**. This developmental pathway is commonly known for its fundamental role in embryogenesis, which aids the cell in differentiation, proliferation and apoptosis. In the absence of Wnt signaling, cytoplasmic β-catenin complexes with the tumor suppressors: adenomatosis polyposis coli (APC) and Axin1, as well as the glycogen synthase kinase-3β (GSK-3β). In this complex, GSK-3β phosphorylates β-catenin, targeting it for ubiquitiniation and subsequent degradation. In the event that Wnt signaling receptors are engaged, conformational changes in the Axin complex cause the release of β-catenin, which then localizes to the nucleus and activates the transcription of Myc, cyclin D1 and COX2 amongst others [57-59]. In HCC, our studies and a number of other transcriptomic and proteomic studies have indicated an increase in Wnt signaling, possibly as a result of an accumulation of *Axin1 *mutations at sites that bind β-catenin and/or *CTNNB1 *mutations along sites marked for phosphorylation by GSK-3β [60,61]. It is hypothesized that an increase in signaling from the Wnt pathway is necessary to maintain "stemness" in HCC, characterized by cell proliferation and immortality, an event that may be representative of CSCs [60,62].

**Myc **is a potent oncogene, which appears to be constitutively up-regulated in many human cancers, representing a phenomenon of "oncogene addiction." Though about 30% of HCC cases show an up-regulation of Myc because of the Wnt/β-catenin pathway[63], its increased expression in HCC is also attributable to the activation of its locus through chromosome amplification [64] One possible mechanism by which Myc contributes to hepatocarcinogenesis is through the induction of telomerase, which also appears to be active during HCC development[65], thereby bypassing cellular senescence. Moreover, the up-regulation of Myc in a variety of tumors has also been associated with deregulated microRNA (miRNA) expression in many human malignancies [66], which as discussed in the next section, have a significant impact on tumorigenesis and progression. On the other hand, the inactivation of Myc in HCC causes a subpopulation of cells to differentiate while the rest remain dormant, giving rise to a phenotypically diverse tumor population and possibly the origin of CSCs [67].

**PI3K/PTEN/Akt**. The activation of the Akt pathway is mediated by either an activated tyrosine kinase receptor, or more rarely the constitutive activation of PI3K or the loss of phosphatase and tensin homolog (PTEN). PTEN is a tumor suppressor gene and the PTEN protein functions as a negative regulator of Akt. The loss of PTEN expression via a loss of heterozygosity in chromosome 10q along with an activation of Akt has been reported in 40%-60% of HCC cases [68,69]. Since Akt is involved in a number of biological processes, such as cell survival, cell growth, apoptosis and differentiation, its deregulation has been implicated in many human cancers. Though the role of Akt in HCC is not confirmed, its activation is interestingly linked to more aggressive tumors in HCC [70] and an activation of β-catenin signaling in intestinal stem cells, suggesting that the two oncogenic pathways: PI3K/PTEN/Akt and Wnt/β-catenin may be interconnected to promote stemness and carcinogenesis [71].

#### Aberrant expression of miRNAs in HCC

In recent years, the aberrant expression of miRNAs has been implicated in a wide variety of human cancers. miRNAs are a class of small non-coding RNAs that play a critical role in biological processes of cell development and differentiation and the deregulated expression of miRNAs in HCC has revealed their functional involvement in HCC carcinogenesis and progression [72].

For example, in HCC cases, gene expression profiling reveals that an up-regulation of miR-181 is associated with increased signaling in Wnt/β-catenin pathways and conversely, siRNA mediated inhibition of the TGF-β pathway indicates a decreased expression of miR-181 [73,74]. Moreover, loss of let-7g expression is associated with HCC metastasis [75]. miR-26 expression has been found to be associated with HCC gender disparity and silencing of miR-26 in tumor cells is linked to a subtype of HCC with an activated inflammatory pathway and a favorable response to interferon therapy [76]. In addition, increased expression of miR-21 has been associated with loss of heterozygosity at the PTEN locus, consequently activating the Akt pathway and promoting tumorigenesis [77,78]. Similarly, miRNAs associated with the cell cycle regulation and apoptosis inhibition in HCC have also been identified [79].

A study in our lab has revealed a 20-miRNA-based signature that is associated with HCC venous metastasis, details of which are expanded upon at the end of this article. This signature provides promise to a future of personalized medicine since it can be used clinically to identify patients with early-stage disease or metastases and can even be used to predict survival and recurrence [80].

### Epigenetic modifications may improve the early detection of HCC cases

In the last decade there has been increasing evidence to support the occurrence of aberrant DNA methylation patterns in human HCC [27]. Therefore, in addition to genetic mechanisms of deletions or mutations, epigenetic changes can increase or decrease gene expression via regulating DNA methylation. DNA methylation in the mammalian genome is found at the cytosine residues of CpG dinucleotides, often associated with promoter-related CpG islands. Though methylation is imperative for normal development and differentiation, aberrant hypomethylation in HCC and many human cancers can lead to the expression of oncogenes [81], or, similarly, hypermethylation can lead to the silencing of tumor-suppressor genes [82]. In HCC, an increased expression of DNA methyltransferases (DNMTs), enzymes which catalyze epigenetic alterations, occurs early in the development of tumorigenesis. The frequency of aberrant DNA methylation increases from precancerous lesions to dysplastic nodules and finally HCC, signifying their important role in tumor progression [83]. For instance, the tumor suppressor genes: *RB1 *[84] and *CDKN2A *[36] have been shown to be hypermethylated in HCC, leading to uncontrolled cell proliferation. Likewise, PTEN promoter methylation has also been reported in HCC, which allows the progression of the PI3K/PTEN/Akt pathway [85].

Epigenetic changes in HCC have also been reported at the miRNA level. For example, the deregulation of miR-1 due to hypermethylation in HCC was reversed with 5-azacytidine, resulting in decreased cell proliferation and increased apoptosis [86]. A similar association has been observed with miR-124 and also miR-203, amongst others, in HCC [87].

Since distinct methylation signatures of tumor suppressor genes have been observed in high-risk subjects up to 9 years before clinical diagnosis, DNA methylation profiling may provide a unique tool to reliably predict cancer status. Apart from their potential as a diagnostic platform though, further understanding of methylation patterns in HCC may provide them useful in determining recurrence and survival, as well [88].

### Transcription profiles and the identification of HCC tumor subtypes

Recent breakthroughs in technology have provided comprehensive genetic and transcriptomic profiling platforms that are successfully used in identifying tumor subtypes and predicting patient outcome and survival. In addition to classifying tumor aggressiveness, high-throughput molecular profiling systems are also useful in determining how the tumor will respond to treatment.

Global gene expression analysis of HCC has revealed two distinctive subclasses of human HCC that are highly associated with patient survival [89]. The low survival subclass was marked by increased expression of cell proliferation and antiapoptotic genes, such as PCNA and PTMA, respectively. Moreover, the poor survival subgroup also expressed a higher number of genes related to ubiquitination and histone modification, namely UBE2D1 and HRMT1L2, respectively.

Additional transcriptome analysis of HCC samples has classified HCC into 6 subgroups associated with clinical and genetic characteristics, which further identify two tumor groups linked to chromosome instability (Groups 1-3) or stability (Groups 4-6) [90]. The first group was linked to low copy number HBV infection, particularly in Africa, increased *Axin 1 *mutations, absence of *TP53 *mutations and possessed an over expression of imprinted genes. The second group was linked to high copy number HBV infection, many regions of LOH, and *TP53 *and *Axin1 *gene mutations. These first two groups are the only ones that employed the Akt biological pathway and appeared most genetically distinct from the remaining subgroups. Groups 5 and 6 were also substantially different since they were easily classified based on the abundance of *CTNNB1 *mutations (near 100%) and the high level of Wnt pathway activation.

Recently, our lab identified another 2 HCC subtypes based on their expression of a hepatic stem cell marker, epithelial cell adhesion molecule (EpCAM) and EpCAM-coexpressed genes[91]. EpCAM-positive HCC correlated with increased Wnt pathway activation, cytokeratin 19 and c-Kit, which are all known markers of progenitor cells. On the other hand, EpCAM-negative HCC resembled gene expression patterns of mature hepatocytes. Further analysis based on AFP levels allowed these two subgroups to be further divided into 4 subtypes with the ability to predict prognosis. Based on this classification, poor clinical outcome is correlated with AFP expression and good prognosis was only associated with EpCAM^+ ^AFP^- ^cases.

Insights into tumor response to treatment are being elucidated and our lab recently identified the importance of miR-26a and miR-26b in survival and response to interferon therapy [76]. Compared to paired noncancerous tissue, HCC samples had decreased miR-26 expression correlating with an increase in nuclear factor κB (NF-κB) and IL-6 signaling. Furthermore, clinical data revealed that those patients with low miR-26 expression had a better response to adjuvant interferon therapy with interferon alpha than those with high miR-26 expression.

Taken together, transcriptional profiling platforms are beginning to provide a vast amount of useful information that can be synthesized to identify HCC subtypes, patient outcome, and survival and treatment options. Still, new insights into the potential cellular origin of HCC and its activated molecular pathways are necessary to improve targeted therapy.

### Two models of hepatocarcinogenesis may complement one another

The longstanding clonal evolution model for HCC development is a multistep event, which may take 30 years to unfold. As described earlier, the various etiological factors, particularly inflammation and viral hepatitis, appear to contribute significantly to approximately 90% of HCC cases by creating phenotypically altered hepatocytes. The stepwise progression from altered hepatocytes to dysplastic nodules, or precancerous lesion, occurs as a consequence of chronic inflammation and genomic alterations, which commonly precede HCC [27,39,92]. Therefore, the accumulation of genetic and epigenetic changes, such as the loss of tumor suppressor genes and the gain of an oncogene, gives rise to a mass of primary tumor cells that are considered monoclonal in origin[92,93]. Though from a scientific standpoint this model has vastly improved our understanding of the various genetic and signaling events underlying HCC development, it is unfortunate that these findings have not been translated into better treatment options as liver resection and transplantation still remain the best choice and only benefits a small population.

**Figure 2:**
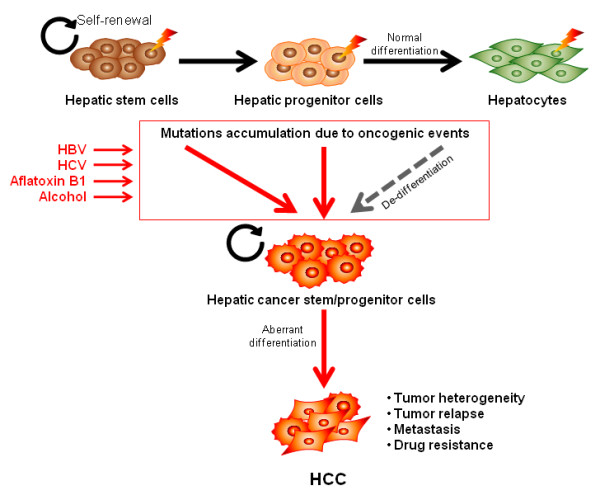
**Cancer Stem Cell Model for HCC Tumorigenesis**. The generation of a CSC model will more effectively benefit the clinical treatment of HCC patients, allowing therapy directed at the most aggressive cells. So far, there is no compelling data demonstrating that HCC follows this model. To test the CSC model, at least two terms have to be addressed: 1) The vast majority of HCC cells, excluding the small subpopulation of CSCs, lack tumorigenic capacity; 2) These CSC populations are distinguished by epigenetic rather than genetic differences because the CSC model argues that CSCs undergo hierarchical differentiation and the epigenetic changes are irreversible. Two HCC subgroups were recently identified based on the expression of AFP and EpCAM. EpCAM^+^AFP^+ ^HCC subgroup (HpSC-HCC) had the features of hepatic stem/progenitor cells and EpCAM^-^AFP^- ^HCC subgroup (MH-HCC) featured as matured hepacytes. HpSC-HCC displayed the ability to self-renew, differentiate and also generate highly invasive HCC. Based on these observations, it is plausible that HCC may represent another solid cancer type that follows the CSC model besides breast, brain and colon cancers. Consistent with the clonal evolution model, HCC CSCs can arise from the mutation of normal hepatic stem/progenitor cells. Though there has not been evidence showing that HCC CSCs can arise from differentiated hepatocytes, the possibility still exits, as there are examples of this in hematopoietic malignancies. Overall, not in contrast to the clonal evolution model, an accumulation of mutations during the normal development of hepatocytes as a consequence of exposure to the various risk factors of HCC might contribute to the rise of the hepatic CSCs; therefore the two models do not contrast but complement each other.

A more recently proposed stem cell model for HCC tumorigenesis may provide a more personalized approach to address diagnostic and therapeutic strategies in the clinic (Figure 2). This model hypothesizes that HCC could be derived from progenitor cells or de-differentiated transformed cells; based on the observation that embryonic stem cells (ESC) and CSCs behave similarly. This would be able to explain the heterogeneous nature of HCC morphology, clinical behavior, and molecular profiles [60,94]. Since the liver is an organ with regenerative capacity, it has bi-potential progenitor cells that can give rise to hepatocytes or chloangiocytes, which could possibly develop into HCC or ICC, respectively [95,96]. Furthermore, cases with a mixed morphology have also been identified. Based on the expression of EpCAM, a substantial number of HCC cases consist of progenitor cells and their heterogeneous progeny with a capacity to self-renew and limitlessly divide [60,91,94]. This relatively new hypothesis is not intended to be contradictory to the step-wise model, but merely complementary in explaining the origin of a more comprehensive group of HCC cases and the arising issues in diagnosis and treatment.

For example, mature hepatocytes, cholangiocytes or bi-potential progenitor cells that acquire mutations through random genetic or epigenetic events can introduce a genetic imbalance in the primary tissue, resulting in the de-differentiation of mature cells and the loss of cell cycle control and/or the ability to continuously self renew. Depending on the extent of genetic alterations, the tumor cells may remain benign or develop and metastasize. Therefore, events initiated by the multistep carcinogenesis model can also result in heterogeneous tumors with stem cell capability and the potential to be more aggressive.

## Intrahepatic Cholangiocarcinoma

An increasing global incidence of ICC [5] has recently hastened research in this field to understand the mechanisms underlying pathogenesis of this dreadful disease. Reviewing the mechanisms of ICC indicates that similarities can be drawn between ICC and HCC, which may improve the prospects of this disease in a clinical setting. Particularly, the tumorigenesis models proposed for ICC development are remarkably similar to those for HCC. Furthermore, several histopathologic and gene expression profiling studies have shown PLC tumors that exhibit a combination of HCC and CCA traits, suggesting an overlap between these tumor types. A subtype of tumors showing combined characteristics of hepatocellular-cholangiocarcinoma (CHC) have been reported and proposed to develop from the bi-potential liver stem cells [97]. Even more recently, a new subtype - cholangiocarcinoma-like HCC (CLHCC) -was discovered and characterized as HCC expressing CCA-like traits. The heterogeneity observed between all 4 tumor subtypes could be indicative of their cellular origins from different developmental stages and may also represent a novel way to approach targeted therapy in CCA and HCC [98].

Comparable to HCC, ICC most commonly arises in the setting of chronic inflammation, often within bile ducts [99] and likely due to liver fluke *Opisthorchis viverrini *infestation [100], PSC[101] or hepatolithiasis[102]. The continuous production of inflammatory cytokines and the induction of inducible nitric oxide synthase (iNOS) lead to oxidative and nitrosative DNA damage [100], increased cell turnover and inhibition of DNA repair mechanisms [103]. In the stepwise model, an accumulation of inflammatory-mediated genetic and epigenetic alterations has been proposed to lead to the successive development of ICC from biliary epithelial cells to biliary dysplastic lesions and eventually cancer [104].

There is growing evidence supporting a hepatic stem cell model of cholangiocarcinoma [96,105,106]. Interestingly, the same bi-phasic progenitor cell can give rise to hepatocytes and cholangiocytes, as mentioned earlier, and each of these cells has longevity and repopulating potential [107]. Therefore, in the setting of chronic inflammation, a tumor could arise from the clonal evolution of either mature cholangiocytes which de-differentiate, or progenitor cells; allowing a combination of the two proposals. Furthermore, depending on the degree of differentiation achieved before maturation arrest, one can observe a heterogeneous tumor with a range of neoplastic phenotypes [107]. One author explains that the dysplastic nodules observed during carcinogenesis may be adaptive non-oncogenic responses to carcinogenic substances, rather than a multistep accumulation of genomic alterations [105].

### Genetic alterations that manifest in ICC

#### Genomic alterations

In a study aimed at distinguishing chromosomal changes between HCC and ICC, CGH analysis reveals the frequency of chromosomal losses in ICC is higher [53]. Short segments of chromosomes 1p [108], 3p, 6q and 9q [28,108] are commonly deleted in ICC, with a frequency of at least 55%, whereas the frequency of such events in HCC are usually less than 40% [28]. Commonly amplified regions in ICC are in segments of 1q, 7q, 7p and 8q [28,108], with an amplification frequency of at least 30%. Losses in regions of 6q and 3p appear to be highly characteristic of cholangiocarcinoma, but overall the high frequency of gains and losses appears to carry a poor prognostic value [28]. Further studies to detect the loss of tumor suppressor genes during a consistent LOH have indicated a high rate of allelic losses at 5q and 17p [109,110]. LOH has been observed in other chromosomal regions, but to a lesser extent, and may represent random error. HCC and ICC share similar allelic losses in the 5q and 17p regions, allowing some to propose that these two tumors arise from the same CSC and therefore may share similar genetic changes during tumorigenesis [105,110]. Persistent structural genomic changes in these cells have been associated with a variety of mutations, conferring a loss in tumor suppression and the amplification of oncongenic pathways.

***TP53 ***mutations in ICC are common and their frequency ranges from 20-80% depending on the geographic region [111,112]. Though the deregulation of p53 in ICC is similar to HCC, as there is a loss of cell cycle control and a decrease in apoptotic events, the deregulation in ICC is sometimes more associated with an accumulation of inactive wild-type p53 and its inhibitor mdm-2, rather than a loss of function mutation [113,114]. This renders the p53 regulatory pathway non-functional and supports the notion that either *TP53 *mutations or an alteration to the p53 pathway may be critical to the development of ICC [115,116]. The regulation of cell cycle entry by p53 involves, amongst others, the p21^WAF1/Cip1 ^protein, which binds to the cell division kinase (CDK) 4:cyclin D complex and prevents the phosphorylation of Rb protein. The CDK4:cyclin D complex is also influenced by the p16^INK4A ^inhibitory protein[117], which coincidentally is altered by LOH and/or promoter hypermethylation in 25%-83% of resected cholangiocarcinoma specimens [118,119].

**K-*ras ***mutations occurring at codon 12 are often observed in ICC, involving either a glycine to aspartic acid or a glycine to cystine transition [111,115]. Less frequent mutations have been observed in codon 13 (second nucleotide) and codon 61 (third nucleotide) [120]. K-*ras *mutations corresponding to over expression are observed early in carcinogenesis, which suggests an important role in ICC development. Furthermore, K-*ras *has been implicated in aggressive ICC downstream of the biliary tree with increased expression levels in metastatic lymph nodes [121]; therefore K-*ras *expression correlates to poor prognosis [122].

#### Transcriptomic alterations: Enhanced proliferation signaling in ICC

The inactivation of tumor suppressor genes and the concordant amplification of proto-oncogenes, such as *TP53 *and K-*ras*, respectively, play a significant role in altering the signaling network and promoting tumorigenesis. The exact mechanism by which these pathways are affected is currently unknown, especially since most of the mechanisms have only been observed in ICC cell lines. Since in vivo studies in animal models poorly correlate with clinical outcome, the need to recreate an ICC model to better understand this disease is underscored. Despite this lack of conclusive data, several studies have identified a selection of important mechanisms contributing to the development of ICC, emphasizing the role of TGF-β, IL-6, STAT-3, COX-2 and β-catenin [99,123]. Because of the similarities already observed in the etiology and structural changes between HCC and ICC, and since some pathways most likely overlap, one may try to infer the mechanisms underlying HCC on ICC and compare this with gene expression arrays to arrive at a more functional understanding of molecular pathogenesis in ICC.

#### Transcriptomic alterations: the recent identification of miRNAs in ICC

Just as in HCC, the altered expression of miRNAs in ICC has been reported to contribute to tumor growth. Malignant cholangiocytes appear to be marked by an over-expression of miR-21, miR-141 and miR-200b [124]. The increased expression of miR-21 and miR-200b has been linked to increased cellular proliferation, mediated by a down-regulation of PTEN and ZFHX1B tumor suppressors, respectively [78,124]. In addition, the decrease in miR-29b has been linked to the increased expression of MCL-1, an anti-apoptotic protein, resulting in decreased apoptosis [125]. Though the network of miRNA involvement is far greater in HCC, the study of miRNAs in ICC is likely to lead to novel diagnostic and prognostic methods once their function is confirmed.

### Future Perspectives

Currently, surgery such as liver resection remains the best treatment option for early HCC and ICC but tumor recurrence is still predominant in about 80% of HCC cases [126] and may be higher in ICC cases [127]. To make matters worse, effective treatment is limited for advanced stage carcinoma, which emphasizes the need to improve our understanding of primary liver cancers and consequently help improve patient diagnosis during the early stage. Intervention early in the process will improve treatment and prognosis, which may be conferred by an attempt at personalized medicine.

Individual genetic background has been suggested to contribute to HCC risk, given that only a fraction of patients with chronic liver disease or PSC actually develop HCC or ICC, respectively, even though greater than 50% of cases occur in the setting of inflammation. For this reason, the identification of genetic susceptibility loci and new biomarkers are essential to improving diagnosis and treatment outcome, and GWAS studies in liver cancer are highly necessitated.

The hypothesized CSC model for the development of HCC and ICC, and the molecular pathways, such as the Wnt/β-catenin pathway and miR-181, provide valuable information about tumor growth and invasiveness. Since recently, the role of EpCAM in maintaining a stem cell phenotype in HCC and ICC is being elucidated but our studies, as well as others provide evidence for its role in the promotion of proliferation, migration and invasion potential in cells with activated Wnt/β-catenin signaling [60,91,128]. Therefore, β-catenin may be a novel target in the prevention of carcinogenesis [57], highlighting the importance of molecular profiling to characterize the population of cells and their distinct molecular pathways [60].

The role of miRNA signatures has recently been elucidated through the examination of miRNA expression profiles of samples from two different subtypes in HCC, indicating miRNA profiling can be used to indicate tumor origin and aggressiveness. In these studies [60,91,129], the two subtypes are hepatic stem cell like HCC (HpSC-HCC; EpCAM^+^AFP^+^) and mature hepatocyte like HCC (MH-HCC; EpCAM^-^AFP^-^), where HpSC-HCC cells are hepatic CSCs with the ability to self-renew, differentiate and initiate aggressive tumors in vivo [60]. Coincidentally, among HpSC-HCC tissues, miR-181 is found to be up-regulated and functions in promoting stemness by targeting hepatic transcriptional regulators of differentiation, such as CDX2 and GATA6, and nemo-like kinase (NLK), an inhibitor of Wnt/β-catenin signaling [73].

The exciting identification of miRNA signatures unique to HCC offers new platforms for cancer diagnosis and prognosis. For instance, in addition to the 20-miRNA-based signature described above that is able to differentiate between CSC-like and mature hepatocyte-like HCC tumors, there are additional miRNAs that can help differentiate between benign and malignant tumors, or between alcohol induced or HCV induced HCC [130]. Prognostic miRNA markers of HCC also exist, and in fact they are specific enough to help with determining metastasis [80], recurrence [131] and survival [75,76,80], independently.

Studies have also indicated the therapeutic potential of miRNAs, particularly with the observance that anti-miR-181 can reduce tumorigenicity in mice with hepatic CSCs, cells that are otherwise resistant to chemotherapy with 5-Fluorouracil (5-FU) [60]. Along with others, our studies indicate that miR-26 functionally acts as a tumor suppressor by inhibiting cell proliferation, and a low miR-26 expression is associated with poor prognosis [76]. miR-26 expression is significantly down-regulated in HCC, but gene therapy with the delivery of miR-26 to hepatocytes considerably blocks Myc-induced HCC [76,132]. Furthermore, studies in our lab on miR-26, NF-κB and IL-6 have revealed the potential of miRNA expression profiles in the stratification of patients for interferon therapy [76].

## Conclusion

The studies of molecular mechanisms involved in the progression to HCC have been investigated at length, and they have helped infer testable hypotheses in ICC. Advances in molecular profiling studies using DNA-microarray based gene-expression profiling have provided increased awareness about the regulatory networks altered in human HCC and have also provided useful gene expression-based signatures that can distinguish tumor subtypes, assist clinical staging and predict patient outcomes. Since molecular profiling is proving to be an efficient way to gain insight into the molecular mechanisms underlying carcinogenesis, these techniques should also be employed more extensively in ICC so that we can obtain a more inclusive picture of regulatory elements in pathogenesis.

Advances in the specificity and sensitivity of molecular profiling platforms including expression analysis and comparative genomics with the additional incorporation of PLC databases and bioinformatics tools, we are approaching a new era for understanding the heterogeneity of HCC and ICC tumors. Such integrated platforms are enabling our improved understanding of the etiology, tumor microenvironment and the carcinogenesis of these two devastating diseases, which we hope to integrate with a personalized approach in improving the clinical outcome of these cases.

## Abbreviations

5-FU: 5-Fluorouracil; AFB: aflatoxin B1; AF: alpha-fetoprotein; CCA: cholangiocarcinoma; CDK: cell division kinase; CGH: comparative genomic hybridization; CSC: cancer stem cells; DNMT: DNA methyltransferases; EpCAM: epithelial cell adhesion molecule; GWAS: genome-wide association studies; HBV: hepatitis B virus; HCC: hepatocellular carcinoma; HCV: hepatitis b virus; HpSC-HCC: hepatic stem cell like HCC; ICC: intrahepatic cholangiocarcinoma; LOH: loss of heterozygosity; MH-HCC: mature hepatocyte like HCC; PLC: primary liver cancer; PSC: primary sclerosing cholangitis; SNP: single neucleotide polymorphism.

## Competing interests

The authors declare that they have no competing interests.

## Authors' contributions

MRK drafted the manuscript, XZ designed the figures and XWW contributed his ideas and helped edit the paper. All authors participated equally in the design and development of this review and all authors read and approve the final manuscript.
